# Dexmedetomidine vs. midazolam at single low doses for opioid and muscle relaxant-free anesthesia in pediatric dentistry: a randomized control trial

**DOI:** 10.3389/fsurg.2026.1914479

**Published:** 2026-09-07

**Authors:** Xiang Zhang, Wei Cui, Dan Qiao, Haichao Sun, Hao Jia, Xuehua Wang, Zhen Ren

**Affiliations:** 1 Department of Anesthesiology, Peking University School and Hospital of Stomatology, Beijing, China; 2 Department of Pediatric Dentistry, The No.2 Hospital of Baoding, Beijing, China; 3 Department of Anesthesiology, The No.2 Hospital of Baoding, Beijing, China; 4 Department of Oral and Maxillofacial Surgery, Fifth Clinical Division, Peking University School and Hospital of Stomatology, Beijing, China

**Keywords:** comparative effectiveness, emergence agitation, pediatric operative dentistry, recovery quality, spontaneous ventilation

## Abstract

**Objectives:**

To verify whether a single low dose of dexmedetomidine could reduce emergence agitation (EA) among pediatric dental patients undergoing outpatient general anesthesia in a clinical dental setting.

**Study design:**

Prospective randomized clinical trial.

**Methods:**

This study included 90 children scheduled for elective dental procedures. Patients were randomly assigned to three groups: midazolam (MID, 0.02 mg/kg, *n* = 30), dexmedetomidine (DEX, 0.2 *μ*g/kg, *n* = 30), and normal saline (NS, *n* = 30). The primary endpoint was EA, with time to extubation (TOE), time to wake-up (TOW), hemodynamic fluctuations and anesthesia-related complications considered as the secondary outcome.

**Results:**

The incidence of EA in the DEX group was significantly lower than that in the saline group, while the incidence in the Mid group showed no significant difference compared with the other two groups after a Bonferroni correction. The three groups showed statistically significant differences in TOW recovery time, with the DEX group having the longest, the Mid group intermediate, and the saline group the shortest.

**Conclusion:**

A single low dose of dexmedetomidine at induction significantly reduces EA in pediatric dental outpatients, without compromising day-surgery discharge feasibility despite a modest prolongation of recovery time.

**Clinical Trial Registration:**

https://www.chictr.org.cn/showproj.html?proj=215673

## Introduction

In pediatric dental clinics, uncooperative children (1) can disrupt the normal progression of treatment, affect the psychological state of other young patients, interfere with the concentration and satisfaction of dental specialists, increase nursing workload and costs, induce parental anxiety and tension, and carry the risk of accidental injury to the child due to operative instruments (2, 3). For such children, our pediatric dental clinic opts for dental treatment under general anesthesia. Of clinical relevance, postoperative emergence agitation (EA) in children is associated with similar issues as mentioned above (2, 4–6), which contradicts the original intent of providing comfortable dental treatment under general anesthesia in developing countries. Current research indicates that the incidence of postoperative EA in elective surgical inpatients receiving inhalational anesthesia alone is as high as 70%–80% (7). However, this rate can be reduced to 4.8%–26% with the combined use of dexmedetomidine during the induction phase (8), to 16.7%–26.5% with midazolam (4), to 2.6% with the combined infusion of propofol during the maintenance phase (9), and to 32% with remifentanil (10).

In most studies on reducing postoperative delirium, the intervention was administered as either an intraoperative continuous infusion (11–13) or a single dose at surgery's end (4, 14), combined with intraoperative opioids. This approach required hospitalization and was associated with prolonged recovery time (15). Most studies did not use the PAED scale—the most validated tool with high sensitivity and specificity—to diagnose or assess delirium severity (16).

Sevoflurane-based, opioid- and muscle relaxant-free anesthesia facilitates rapid emergence, meeting the requirements of pediatric dental day surgery in our hospital, where patients are typically discharged within two hours (9). Under this protocol, EA is a well-recognized challenge, and dexmedetomidine's central α_2_-agonism is ideally suited to mitigate this, provided it is active during the emergence period.

Dexmedetomidine acts on the locus coeruleus to produce arousable sedation mimicking non-rapid eye movement (NREM) sleep. It also suppresses nociceptive transmission, reducing sevoflurane-induced hyperexcitability. Its rapid redistribution and lack of respiratory depression facilitate early extubation. These properties make a single induction bolus ideal for brief pediatric dental procedures: effective during surgical stimulation, yet allowing timely recovery within the day-surgery window (17).

For brief pediatric dental procedures, a bolus given at induction ensures that peak plasma concentrations coincide with the most stimulating surgical phases, while its declining concentration towards the end of surgery permits timely recovery—a critical advantage over continuous infusions (13).

Literature on the use of a single low dose of dexmedetomidine (0.2 μg/kg) in pediatric dental outpatients undergoing sevoflurane-based, opioid- and muscle relaxant-free general anesthesia remains scarce. In particular, no prior study has directly compared this specific regimen against midazolam — the current standard of care — in this setting. Given that sevoflurane-based anesthesia without opioids is associated with a high incidence of EA (7), and that EA can lead to patient injury, prolonged recovery, and parental anxiety, we identified a clear need for an alternative sedative strategy that reduces EA without delaying discharge. We therefore hypothesized that a single low dose of dexmedetomidine administered at induction significantly reduces the incidence of EA compared with midazolam, while preserving rapid recovery in this outpatient population.

### Materials and methods

This prospective, single-site RCT was approved by the Ethics Committee of Baoding Second Hospital (KY2023045), registered at www.chictr.org.cn (ChiCTR2400080125), and conducted in accordance with the Declaration of Helsinki and CONSORT guidelines (Supplementary File 1), with written informed consent obtained from all participants' guardians.

### Study population

Eligibility screening occurred one day before surgery. Inclusion criteria: age >3 years, dental procedure lasting 2–3 h under opioid- and muscle relaxant-free anesthesia. Exclusion criteria: American Society of Anesthesiologists (ASA) class > II; allergy to study drugs; severe sleep apnea (index ≥5.0); renal/hepatic insufficiency; long QT syndrome; congenital, developmental, or psychiatric disorders; or clinical trial participation within 3 months.

### Randomization and binding

Subjects were randomized 1:1:1 using a computer-generated allocation sequence with sequentially numbered, opaque, sealed envelopes prepared by a third-party statistician not involved in the trial. On the day of surgery, an independent study nurse opened the envelope, prepared the assigned study drug — 5 mL of normal saline with or without midazolam (0.02 mg/kg) or dexmedetomidine (0.2 *μ*g/kg) (13, 14, 18)— and delivered it to the operating room in a concealed syringe. The anesthetist (Dan Qiao), who was responsible for drug administration, remained blinded to the group allocation throughout the procedure. Postoperative outcome assessments were performed by another anesthetist (Xiang Zhang), also blinded to group allocation.

### Anesthesia procedure and data collection

The patients fasted for 8 h and did not receive premedication. Prior to entering the operation room, their height, weight, and body temperature were measured. Anxiety was assessed by a blinded observer when the patients were separated from their parents by using the Parental Separation Anxiety Scale (PSAS) (17). Patients were monitored for electrocardiography (ECG), pulse oximetry, noninvasive arterial blood pressure (NIBP), axillary temperature and end-tidal carbon dioxide (ET_CO₂_). End-tidal anesthetic gas (ETAG) on the anesthesia machine was used for decreasing anesthesia awareness in patients (19).

All patients received a standardized opioid- and muscle relaxant-free anesthetic protocol. Induction was achieved with sevoflurane (up to 8% ET_sevo_) in 100% O₂ via facemask[the mask acceptance scale (MAS) assessed] (20). Following loss of consciousness, an IV line was placed, and dexamethasone (0.10 mg/kg) plus titrated propofol (1–3 mg/kg) were given until spontaneous breathing ceased, allowing controlled ventilation without neuromuscular blockers. Nasotracheal intubation was then performed. Anesthesia was maintained with sevoflurane (1%–2% ET_sevo_) and propofol (2–4 mg/kg·h), with spontaneous respiration targeted to ET_CO₂_ 45–55 mmHg throughout. All masked study drugs were administered by the blinded anesthesiologist at surgery onset.

Dental procedures were recorded. Local anesthesia (primacaine with epinephrine) preceded painful stimulation; a rubber dam minimized oropharyngeal water/debris accumulation. Standardized institutional protocols were followed for both anesthesia and dental care. The digital anesthesia system captured intraoperative data: ET_CO₂_, tidal volume (VT), respiratory rate (RR), hemodynamics, surgery/anesthesia duration, medications (types/doses), fluid balance, and procedural details.

Propofol and sevoflurane were halted 5 min pre-completion. Extubation criteria included ET_sevo_ < 0.1% along with facial grimacing, adequate VT, eye opening, coughing with mouth open, or purposeful movements. Patients recovered in the lateral position with oxygen and SpO₂ monitoring until conscious (Aldrete >10) (21), and were discharged 2 h postoperatively as our institutional practice.

Postoperative data analyzed included time to extubation (TOE), time to wake-up (TOW), hemodynamic parameters, EA, the Face, Legs, Activity, Cry, and Consolability (FLACC) scale, postoperative nausea and vomiting (PONV), epistaxis, laryngeal spasm (LS), and airway obstruction (AO). The sleep quality, daily activity, and parental satisfaction were assessed via telephone at 24 h post-surgery.

PAED assessments (6, 16) were performed at 0, 5, 15, and 30 min after tracheal extubation in the post-anesthesia care unit (PACU) by a trained nurse who was blinded to group allocation. The assessment was conducted only when the child was awake (defined as spontaneous eye opening to verbal command or, if unresponsive to voice, to gentle tactile stimulation). If the child was asleep at the scheduled time point, the assessment was deferred for up to 2 min; if the child remained asleep with no agitation behaviors, a PAED score of 0 was assigned for that time point. For children who exhibited agitation behaviors (e.g., thrashing, crying, or restless movements) even with closed eyes, the PAED items were scored based on observable behaviors, consistent with the scale's validation guidelines. EA was diagnosed at PAED ≥10, and considered severe if ≥16 lasting >2 min. Rescue propofol (1 mg/kg IV) was given if the child could not be comforted.

Postoperative pain was evaluated using the FLACC scale at 0, 5, 15 and 30 min after full awakening (22). Rescue analgesia (ibuprofen oral suspension 5 mg/kg) was given if FLACC >3. PONV was graded using the Baxter retching faces (BARF) scale (23) until discharge: 0 = none, 2–4 = mild, 6–8 = severe, 10 = vomiting (with nausea/vomiting defined as scores 6–8 and 10, respectively). Telephone follow-up at 24 h assessed sleep (0 = well, 1 = night crying, 2 = intermittent), activity (0 = normal, 1 = depressed but active, 2 = irritable/inactive), and parental satisfaction (11-point, 0–10, 10 = highest). Epistaxis was graded as follows: 0 = none (no blood on tube or posterior pharyngeal wall); 1 = mild (blood on tube or wall); 2 = moderate (pooling on wall); 3 = severe (large pharyngeal blood impeding intubation, requiring urgent orotracheal conversion) (24).

### Outcome assessment

The primary outcome was EA incidence. Secondary outcomes included: TOE (treatment end to extubation), TOW (extubation to eye opening), delayed emergence (>30 min from PACU entry to eye opening/command response), FLACC scores, adverse events (monitored until discharge), epistaxis, and 24 h postoperative sleep quality, daily activity, and parental satisfaction. Adverse events comprised hypotension/ hypertension (>20% mean arterial pressure change from baseline for >5 min), bradycardia (heart rate <70 bpm for >5 min), LS, PONV, desaturation (SpO₂ < 95% for >30 s), and hypoxemia (SpO₂ ≤ 90% for ≥10 s).

### Hypothesis statement and calculation of sample size

The sample size was calculated using PASS software (version 21.0; NCSS, LLC, Kaysville, UT, USA) with a two-sided chi-square test for multiple independent proportions (2 degrees of freedom), setting alpha at 0.05 and power at 90%. The anticipated event rates were derived from previously published studies that were methodologically comparable to our trial population (13, 14). Based on these estimates, the incidence of emergence agitation was assumed to be 60.8% in the saline group, 16.7% in the midazolam group, and 26% in the dexmedetomidine group. The calculated sample size was 80 patients in total. To allow for balanced group allocation and to account for a 10% dropout rate, we rounded up to 90 patients (30 per group), which exceeded the minimum requirement.

### Statistical analysis

The primary analysis was conducted on an intention-to-treat (ITT) basis, including all randomized patients in their assigned groups regardless of protocol deviations or missing data. Normality of continuous variables was assessed using the Shapiro–Wilk test. Normally distributed variables were expressed as mean ± SD and compared among the three groups using one-way analysis of variance (ANOVA); homogeneity of variances was evaluated using Levene's test. When the overall ANOVA was significant (*p* < 0.05), *post hoc* pairwise comparisons were performed using Tukey's honest significant difference (HSD) test if variances were equal, or Tamhane's T2 test if variances were unequal. If the overall ANOVA was not significant, no further *post hoc* analyses were conducted.

Non-normally distributed continuous variables and ordinal variables (e.g., FLACC scores) were expressed as median (IQR) and compared using the Kruskal–Wallis test. When the overall test was significant, *post hoc* pairwise comparisons were performed using Dunn's test with Bonferroni correction. For FLACC scores measured at multiple time points, the Bonferroni correction was applied across these repeated assessments, with statistical significance set at *p* < 0.0167 (0.05 ÷ 3) for each time-point comparison.

Categorical variables were compared among the three groups using the chi-square test, or Fisher's exact test when expected cell frequencies were < 5. When the overall test was significant, pairwise comparisons were performed using the chi-square or Fisher's exact test with Bonferroni correction; the threshold for statistical significance was set at *p* < 0.0167 (0.05 ÷ 3). *P* values were not calculated for outcomes with zero events in all groups.

Missing outcome data were handled using multiple imputation with 5 imputations, assuming missing at random; a complete-case sensitivity analysis was performed to validate the ITT results. A per-protocol analysis was additionally conducted to test the robustness of the findings.

All statistical analyses were performed using SPSS version 26.0 (IBM Corp., Armonk, NY, USA). All tests were two-sided, and a p-value of < 0.05 was considered statistically significant, except where Bonferroni correction was applied as specified above.

## Results

**Figure 1:**
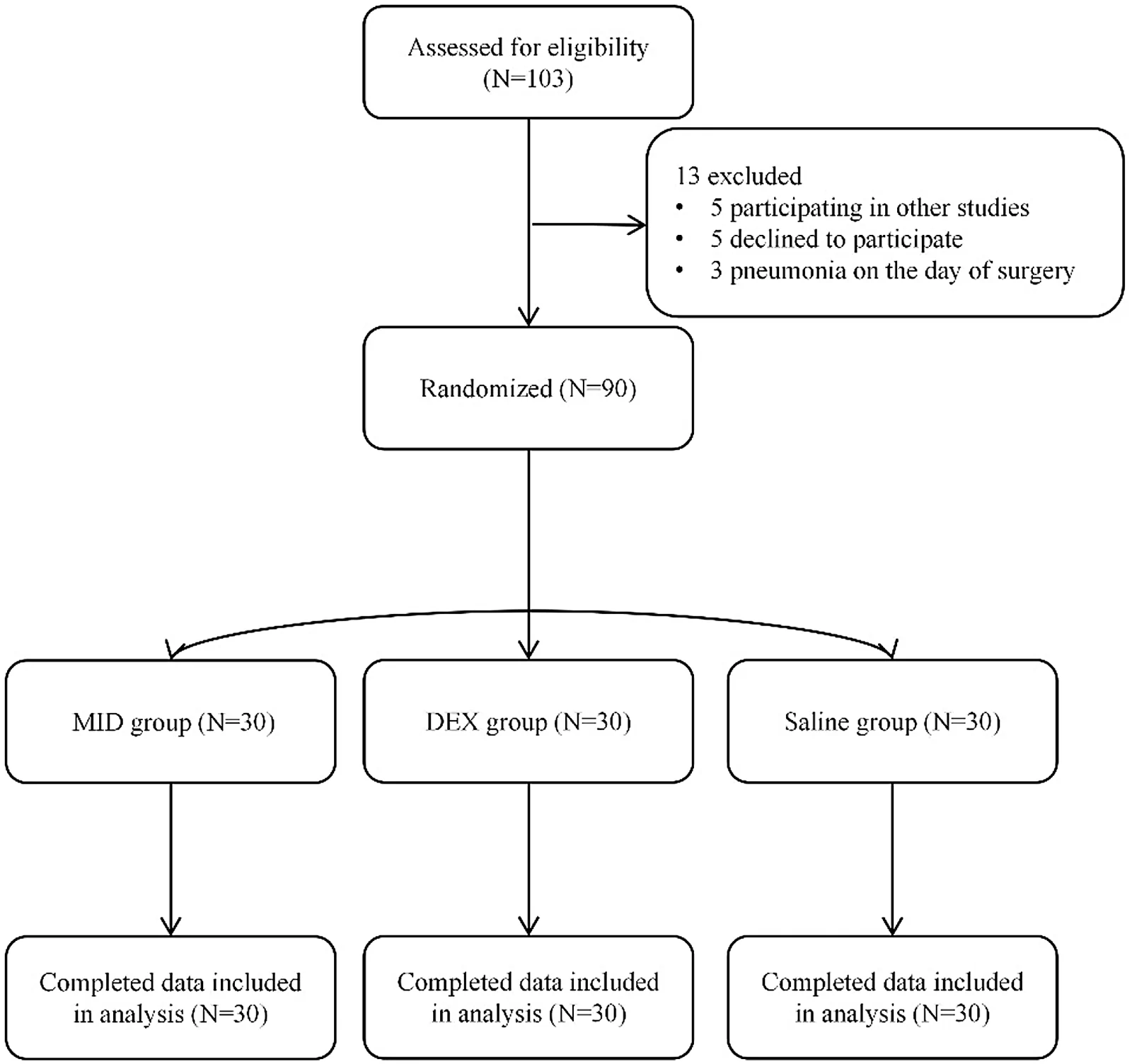
A flowchart of trial.

The present RCT was conducted from May 2024 through May 2025 and enrolled 90 subjects. A flowchart of the trial is presented in Figure 1.

Patient characteristics are shown in Table 1. There were no significant differences in the preoperative characteristics and intraoperative data of patients in the three groups (Table 1).

**Table 1:** Patient characteristics.

Variable	MID group (*N* = 30)	DEX group (*N* = 30)	Saline group (*N* = 30)	*P*
**Preoperative characteristics**				
Age (years)	5 (3, 6)	4 (3, 5)	5 (3, 7)	0.545^§^
Sex (male/female)	20/10	22/8	18/12	0.549^‡^
Height (cm)	99.5 (90.5,112.5)	101 (90.0,113.5)	100 (89.5,117.5)	0.735^§^
Weight (kg)	21.10 ± 4.13	18.67 ± 3.67	19.65 ± 4.51	0.172^§^
BMI (kg/m^2^)	15.26 (13.98,17.09)	15.66 (13.13,16.93)	16.01 (13.42,17.32)	0.641^§^
ASA I/II	30/0	25/5	28/2	0.053^‡^
Baseline HR (beats/min)	103 ± 7	110 ± 12	98 ± 11	0.815^§^
Baseline MAP (mmHg)	68 ± 5	75 ± 3	71 ± 5	0.589^§^
PSAS score	1.5 ± 1.0	1.4 ± 0.9	1.5 ± 1.0	0.812^†^
MAS score	1.8 ± 1.2	2.2 ± 1.5	2.0 ± 1.4	0.584^†^
**Intraoperative data**				
Duration of dental procedure (min)	151.9 ± 9.8	149.2 ± 10.3	150.5 ± 9.3	0.707^§^
Duration of anesthesia (min)	163.5 ± 7.1	159.3 ± 11.2	161.2 ± 6.4	0.639^§^
Time from induction to dental procedure (min)	6.2 ± 0.5	6.5 ± 1.0	6.0 ± 0.7	0.813^§^
Total propofol dose (mg/kg)	7.8 ± 0.6	7.5 ± 0.5	8.0 ± 0.7	0.720^§^
End-tidal sevoflurane (%)	1.8 ± 0.3	1.9 ± 0.3	1.8 ± 0.4	0.410^§^
Dose of local anesthesia (mg)	29 ± 4	29 ± 5	30 ± 4	0.509^§^
Total volume of fluid administered (mL)	280 ± 20	300 ± 15	300 ± 15	0.643^§^
Hypotension	3 (10%)	6 (20%)	4 (13%)	0.533^‡^
Hypertension	0 (0%)	0 (0%)	0 (0%)	—
Bradycardia	0 (0%)	0 (0%)	0 (0%)	—
Number of dental procedures				
Extraction	1.9 ± 0.8	1.9 ± 0.7	2.0 ± 0.8	0.738^†^
Pulpectomy	2.6 ± 1.1	2.7 ± 1.2	2.6 ± 1.2	0.601^†^
Pulpotomy	3.5 ± 0.9	3.1 ± 1.3	2.9 ± 1.1	0.404^†^
Stainless steel crown	2.5 ± 1.7	3.0 ± 1.2	2.6 ± 1.8	0.632^†^
Tooth filling	5.3 ± 2.4	5.5 ± 2.9	5.2 ± 3.0	0.541^†^
Pit and fissure sealant	3.5 ± 0.8	3.3 ± 1.2	3.0 ± 1.0	0.738^†^
Space maintainer (Yes/No)	15/15	10/20	16/14	0.249^‡^

BMI, body mass index; ASA, American Society of Anesthesiologists; MID, midazolam; DEX, dexmedetomidine; HR, heart rate; MAP, mean arterial pressure; PSAS, Parental Separation Anxiety Scale; MAS, mask acceptance scale; SD, standard deviation. Data are presented as mean ± SD, median (interquartile range), or frequency (%).^—^, *P* values were not calculated for outcomes with zero events in all groups.

† Kruskal–Wallis test.

‡ Chi-square test.

§ One-way ANOVA.

Fisher's exact test.

The incidence of EA in the DEX group was significantly lower than that in the saline group, while the incidence in the Mid group showed no significant difference compared with the other two groups after a Bonferroni correction. In all children who developed EA, the episode was successfully terminated following one rescue dose of propofol. There were statistically significant differences in TOW among all three groups: the DEX group had the longest recovery time, the Mid group an intermediate time, and the saline group the shortest (Table 2). Allow spontaneous awakening during the 2-hour recovery stay, no artificial arousal needed.

**Table 2:** Trial outcomes (postoperative data).

Variable	MID group (*N* = 30)	DEX group (*N* = 30)	Saline group (*N* = 30)	*P* (intergroup)	Post hoc pairwise comparisons between groups
Incidence of EA	2	0	8	0.003^¶^	MID_ab_ DEX_b_ Saline_a_（Bonferroni correction）
TOE (min)	4.3 ± 2.7	4.4 ± 2.2	5.1 ± 2.0	0.661^§^	—
TOW (min)	9.1 ± 2.4	14.5 ± 3.3	4.7 ± 1.1	< 0.001^§^	MID vs. DEX: < 0.001; MID vs. Saline: < 0.001; DEX vs. Saline: < 0.001
delayed emergence	0	0	0	—	
FLACC scores in PACU					
10 min	3 (1, 4)	1 (1, 2)	3 (0, 5)	0.746^†^	—
20 min	2 (0, 3)	0 (0, 1)	3 (0, 3)	0.681^†^	—
30 min	0 (0, 1)	0 (0, 1)	1 (0, 2)	0.705^†^	—
Hypotension	0	0	0	—	
Hypertension	0	0	0	—	
Bradycardia	0	0	0	—	
Laryngeal spasm	0	0	0	—	
PONV	0	0	1	1.000^¶^	—
Desaturation	0	0	0	—	
Hypoxemia	0	0	0	—	
Epistaxis after extubation	1	0	1	1.000^¶^	—

Values with different subscript letters in the same row indicate significant differences (Bonferroni-corrected pairwise z-test, *p* < .0167).—, *P* values were not calculated for outcomes with zero events in all groups.

† Kruskal–Wallis test.

§ One-way ANOVA.

¶ Fisher's exact test.

Chi-square test.

No statistically significant differences were observed among the three groups in TOE, FLACC scores, postoperative vital signs, anesthesia-related complications (LS, PONV, epistaxis), sleep quality, or daily activity. Parental satisfaction was uniformly high and did not differ significantly among the three groups (*P* = 0.779). All pediatric patients were safely discharged two hours postoperatively.

## Discussion

Our results demonstrate that a single low dose of dexmedetomidine administered at induction significantly reduces the incidence of EA in pediatric dental outpatients undergoing sevoflurane-based, opioid- and muscle relaxant-free general anesthesia, with a modest and clinically acceptable prolongation of wake-up time that remains well within the predefined discharge criteria for day-surgery procedures.

Zhou et al. determined that the 90% effective dose (ED90) of a continuous intraoperative dexmedetomidine infusion for preventing EA in children aged 3–7 years undergoing dental rehabilitation with sevoflurane anesthesia was 0.74 *μ*g/kg/h (95% CI, 0.67–1.05 *μ*g/kg/h) (17). Our finding does not contradict the ED90 reported by Zhou et al. Instead, the two studies address different research questions and complement each other. Zhou et al. used a biased coin up-and-down sequential allocation method to determine the dose required to prevent EA in 90% of children, providing a precise pharmacologic estimate of potency, and intentionally omitted a loading dose to avoid hypotension or bradycardia. Our study, in contrast, was a randomized controlled trial that tested a fixed, relatively low bolus dose (0.2 *μ*g/kg) in a broader pediatric surgical population and found it clinically effective.

One possible explanation for the apparent efficacy of our lower bolus regimen is that a single induction bolus may produce a transient peak plasma concentration that exceeds the steady-state level achieved by a 0.74 *μ*g/kg/h infusion, potentially compensating for the lower total dose. However, we emphasize that this pharmacodynamic interpretation remains speculative, as we did not measure plasma dexmedetomidine concentrations or perform formal pharmacokinetic-pharmacodynamic modelling in our study. It is also plausible that the lower dose was sufficient in our cohort due to differences in surgical invasiveness, pain intensity, anesthetic management, or patient characteristics — variables that were not directly investigated and therefore cannot be confirmed from our data. Taken together, these observations generate the hypothesis that a single low bolus may be adequate in selected clinical settings, but this hypothesis requires direct validation in future studies with appropriate dose-ranging and pharmacokinetic designs. Until such data are available, we caution against assuming pharmacodynamic equivalence between bolus and infusion regimens, and we reinforce the importance of individualizing dexmedetomidine dosing based on the type of surgery, the level of stimulation, and the desired balance between efficacy and safety (e.g., avoiding bradycardia or prolonged recovery).

Dexmedetomidine, a relatively selective α_2_-agonism, possesses sedative, analgesic, and anxiolytic effects, and its intraoperative administration reduces anesthetic requirements, accelerates postoperative recovery, and blunts the sympathetic nervous system response to surgical stimulation (25–28). Shukry et al. concluded that a continuous perioperative infusion of dexmedetomidine at 0.2 *μ*g·kg⁻^1^·h⁻^1^ significantly reduces the incidence of EA from 60.8% down to 26% in children after sevoflurane-based general anesthesia (13). Our findings are consistent with this direction of effect, although the EA incidence in our dexmedetomidine group was lower. This difference may be explained by several factors, lthough these remain speculative as they were not directly investigated in our study. Possible contributing factors include differences in dosing strategy (bolus vs. continuous infusion), the use of propofol as an adjunctive agent in our sevoflurane regimen, and variations in patient population and surgical invasiveness. Regarding the use of propofol, a meta-analysis has shown that propofol administered via continuous infusion can prevent EA in pediatric patients (29), an effect that may be attributable to its rapid pharmacokinetics, antiemetic properties, and ability to allow quicker recovery without residual anesthetic effects (30, 31). However, the individual contribution of propofol to the lower EA incidence observed in our study cannot be determined from our data. We caution against attributing our findings to any single factor, and emphasize that these interpretations remain hypotheses requiring prospective validation.

A statistically significant prolongation of TOW was observed in the dexmedetomidine group, consistent with its longer elimination half-life (26, 28). Importantly, all patients in all groups met the predefined discharge criteria within the 120 min institutional window, and no patient required unplanned hospital admission due to delayed awakening. Thus, while the difference was statistically significant, it did not translate into a clinically meaningful delay in discharge. From a pharmacokinetic perspective, this modest prolongation reflects the expected duration of action of a single bolus of dexmedetomidine and does not represent an unexpected adverse event.

We acknowledge that this represents a trade-off: the longer TOW is the price of the substantially reduced incidence of EA observed in the dexmedetomidine group. However, we argue that this trade-off is clinically justifiable in the specific context of pediatric dental outpatients for several reasons. First, a calmer, more gradual emergence may reduce the need for physical restraint, lower the risk of self-injury or dislodgement of intravenous lines, and decrease parental anxiety — all of which are important quality indicators in pediatric anesthesia. Second, the predictability of the recovery profile (all patients discharged within 120 min) allows for efficient operating theatre scheduling, as the modest prolongation is consistent and foreseeable rather than erratic. Third, for uncooperative or anxious children, a slightly slower but smoother emergence may actually enhance patient safety and caregiver satisfaction, as it avoids the distress associated with acute agitation during awakening (32).

Nevertheless, we acknowledge that in a different clinical setting — such as a high-volume day-surgery unit with extremely tight turnover schedules, or in procedures longer than those in our cohort — even this modest prolongation might become operationally significant. We have therefore expanded our limitations section to emphasize that our findings are specific to brief pediatric dental procedures under opioid- and muscle-relaxant-free anesthesia, and that generalization to other surgical contexts should be made with caution. Future studies incorporating cost-effectiveness or operational efficiency analyses would be valuable to determine whether the benefits of reduced agitation outweigh the modest prolongation of recovery in various healthcare systems.

Consistent with a systematic review of six studies, which reported that dexmedetomidine can be safely administered to neonates within specific dosage ranges without causing significant adverse events necessitating abrupt discontinuation (33)，our study found no statistically significant differences among the three groups in terms of TOE, FLACC scores, postoperative vital signs, anesthesia-related complications (including laryngeal spasm, PONV, and epistaxis), sleep quality, daily activity, or parental satisfaction. Moreover, all pediatric patients in our study were safely discharged two hours postoperatively, further supporting the favorable safety profile of dexmedetomidine observed in the prior review. Another reason is supported by a randomized double-blind crossover trial, in which opioid-free anesthesia significantly reduced the incidence of postoperative nausea and vomiting (PONV) within 48 h compared with opioid-based anesthesia, with rates decreasing from 23% to 5%. Furthermore, the severity of PONV and the incidence of hypotension were also significantly lower in the opioid-free group (34).

Several limitations of this trial should be acknowledged. First, this was a single-center study, and while our anesthetic and discharge protocols were standardized, practice patterns may vary across institutions, potentially affecting the external validity of our findings. Second, the relatively modest sample size was adequately powered for the primary outcome but may have been insufficient to detect clinically important differences in secondary outcomes or to perform meaningful subgroup analyses. Third, our study population was restricted to pediatric patients undergoing minor dental procedures under opioid- and muscle-relaxant-free general anesthesia; thus, our results may not generalize to more invasive surgical procedures, longer operations, or settings where opioid analgesia or neuromuscular blockade is routinely employed. Fourth, the dosing and safety profile of dexmedetomidine in other surgical contexts or under conditions of markedly prolonged surgery remain to be further elucidated. Finally, the lower incidence of EA observed in the dexmedetomidine group may have been influenced by unmeasured confounding factors — such as differences in surgical invasiveness, pain intensity, or anesthetic co-interventions — that were not directly assessed in this trial. These variables represent potential effect modifiers that should be systematically evaluated in future multicenter studies with larger sample sizes and broader surgical populations.

## Conclusion

A single low dose of dexmedetomidine at induction significantly reduces emergence agitation in pediatric dental outpatients, without compromising day-surgery discharge feasibility despite a modest prolongation of recovery time.

## Data Availability

The original contributions presented in the study are included in the article/Supplementary Material, further inquiries can be directed to the corresponding author.
